# A Comprehensive Review of Lyme Disease: A Focus on Cardiovascular Manifestations

**DOI:** 10.7759/cureus.60821

**Published:** 2024-05-21

**Authors:** Michael Wu, Sophia Mirkin, Marissa N McPhail, Hassaan Wajeeh, Stephanie Nagy, Marie Florent-Carre, Cyril Blavo, Michelle Demory Beckler, Kayvan Amini, Marc M Kesselman

**Affiliations:** 1 Osteopathic Medicine, Nova Southeastern University Dr. Kiran C. Patel College of Osteopathic Medicine, Fort Lauderdale, USA; 2 Osteopathic Medicine, Nova Southeastern University Dr. Kiran C. Patel College of Osteopathic Medicine, Plantation, USA; 3 Public Health, Nova Southeastern University Dr. Kiran C. Patel College of Osteopathic Medicine, Fort Lauderdale, USA; 4 Public Health, Nova Southeastern University Dr. Kiran C. Patel College of Osteopathic Medicine, Clearwater, USA; 5 Microbiology and Immunology, Nova Southeastern University Dr. Kiran C. Patel College of Allopathic Medicine, Fort Lauderdale, USA; 6 Cardiology, Nova Southeastern University Dr. Kiran C. Patel College of Osteopathic Medicine, Fort Lauderdale, USA; 7 Rheumatology, Kiran C. Patel College of Osteopathic Medicine, Nova Southeastern University, Fort Lauderdale, USA

**Keywords:** clinical diagnosis of lyme disease, review of literature, atrial fibrillation lyme, tick-borne infections, lyme carditis, lyme's disease

## Abstract

Lyme disease is a tick-borne illness known for its ability to cause multi-systemic manifestations. It can affect several different systems, including neurological, musculoskeletal, and dermatological systems. However, one of the most concerning biological systems affected is the cardiac system. Lyme carditis typically presents with varying degrees of atrioventricular (AV) block. Additionally, current literature also endorses atypical manifestations, including but not limited to atrial fibrillation and bundle branch blocks. These atypical manifestations are important as they can be the first presenting symptoms in patients with Lyme disease. Therefore, educating clinicians on various signs, symptoms, and manifestations of Lyme carditis remains paramount in reducing morbidity and mortality. We conducted a literature review using PubMed, MEDLINE, and CINAHL, collecting a total of 13 articles to gather information on atypical manifestations of Lyme carditis. This literature review serves to summarize the current research and studies describing these cardiac manifestations and the cardiac pathophysiology associated with Lyme disease. These findings aim to contribute to the expanding understanding of Lyme carditis, subsequently preventing long-term effects through prompt diagnosis and treatment.

## Introduction and background

Lyme disease is considered a multi-system, bacterial infection caused by six species in the spirochete family *Borreliaceae*. In North America, *Borrelia burgdorferi (B. burgdorferi)* and, less commonly, *Borrelia mayonii* are the main causative agents of the disease in humans. In Europe and Asia, infection is primarily with *Borrelia afzeli* or* Borrelia garinii* and, less commonly, with *B. burgdorferi, Borrelia spielmanii,* and *Borrelia bavariensis *[1]. Table 1 displays the geographical distribution of the four different *Ixodes* species.

**Table 1:** Geographical distribution of the 4 Ixodes spp. that spread Borrelia spp. Information gathered from [2-5]

Species of Tick	Most Common Locations
Ixodes scapularis	Northeastern states of the United States and Southeastern provinces of Canada
Ixodes pacificus	Northern California, Western Canada most commonly British Columbia
Ixodes ricinus	Widespread throughout Europe from Portugal to Russia, North Africa, Scandinavia
Ixodes persulcatus	Northern Europe, Western Russia, and Northern China

Approximately 476,000 individuals progress from *Borrelia* spp. infection to Lyme disease annually in the United States [6]. Infection typically occurs from the bite of an infected tick of the genus *Ixodes*. *B. burgdorferi* is spread primarily by the black-legged tick in the northeastern, mid-Atlantic, and north-central United States, and by the western black-legged tick in the Pacific Coast states [6]. Table 2 displays the areas of high and low incidence of Lyme disease in the United States.

**Table 2:** Level of incidence rates of Lyme disease in the United States Information gathered from [7]

Incidence Rate	States Affected
High incidence of Lyme disease	Maine, New Hampshire, New York, New Jersey, Pennsylvania, Maryland, Wisconsin, Minnesota
Low incidence of Lyme disease	North Dakota, Michigan, Iowa, Illinois, Indiana, Ohio, Virginia

**Figure 1:**
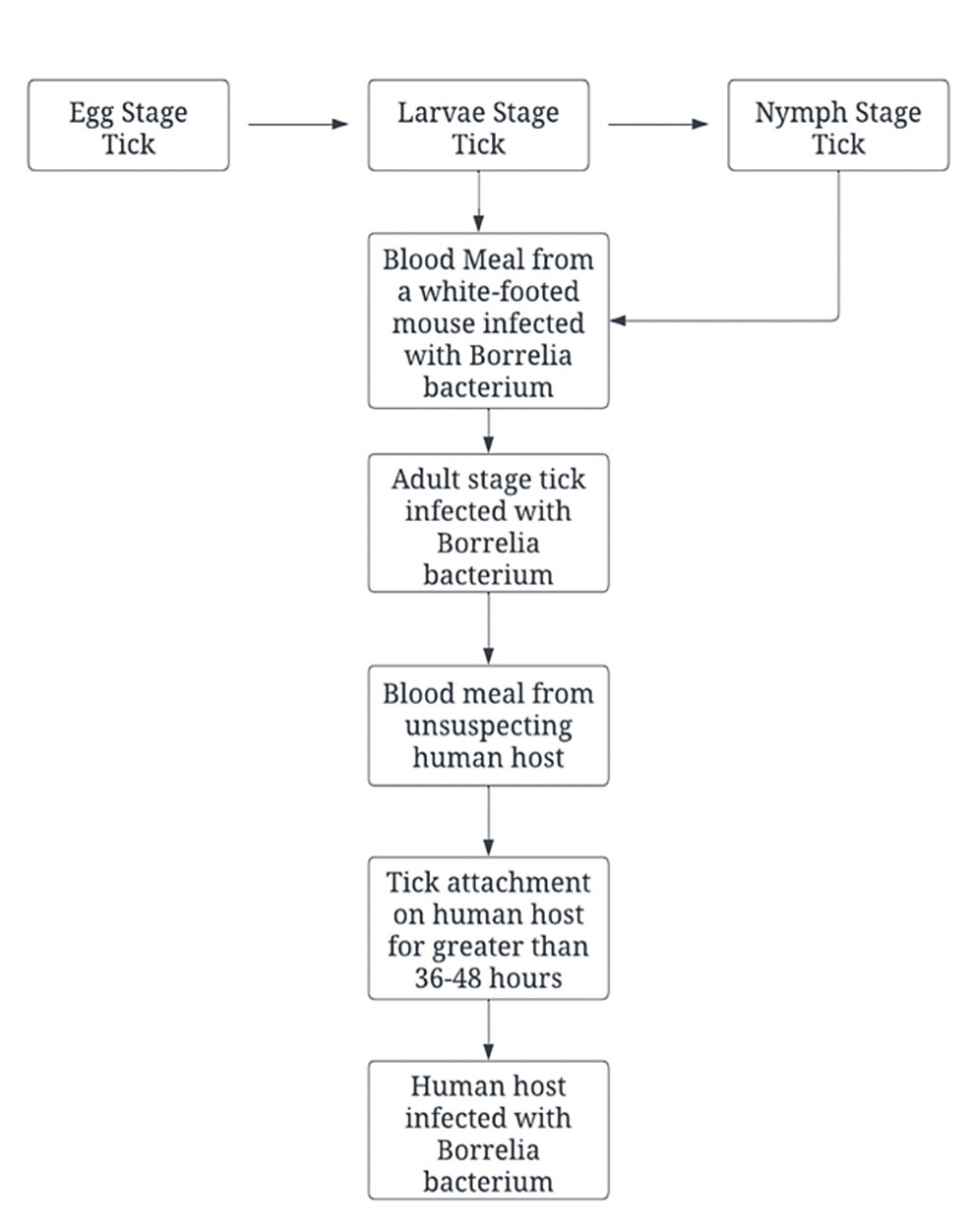
Borrelia spp.-infected tick life cycle Information gathered from [8]

Black-legged ticks undergo four life stages (egg, larva, nymph, and adult) during their two to three-year life cycle. Figure 1 displays the full life cycle of a *Borrelia-*infected tick.

The Centers for Disease Control and Prevention (CDC) recommends the use of clothing containing 0.5% permethrin, insect repellents, and avoidance of wooded and brushy areas with high grass to reduce one’s risk of infection with *Borrelia* and consequent development of Lyme disease [4]. Additionally, it is recommended to conduct a full body check upon return from potentially tick-infested areas including under the arms, the ears, inside the umbilicus, behind the knees, in and around the hair shaft, between the legs, and the waist. A thorough inspection of the body is imperative, as failure to identify the tick in a timely manner allows for *Borrelia* spp. infection and the progression of subsequent systemic manifestations of Lyme disease.

A review of systemic manifestations and current treatments of Lyme disease 

Lyme disease results from a multiorgan bacterial infection that can evolve and progress in clinical presentation among untreated patients. Specific timelines of Lyme disease vary, but consensus among clinicians currently divides Lyme disease into two time categories: acute localized stage and disseminated stage, both of which are divided into early and late disseminated phases. Most commonly, Lyme disease is known for its characteristic skin manifestations that become apparent chronologically within these three stages.

The acute localized stage of Lyme disease is initiated with prodromal flu-like symptoms and a pathognomonic rash called erythema migrans. The lesion of erythema migrans, also known as the bullseye target rash, appears annular and erythematous with central clearing [9,10]. This rash is rarely painful or pruritic; however, it may present with regional lymphadenopathy and spread with disease progression. The rash can progress to an early disseminated cutaneous phase consisting of borrelial lymphocytoma, a small induration that develops into a solitary blue and red hue nodule most commonly on the lip and breast [11]. In advanced cases of untreated Lyme disease, the skin manifestations will progress to acrodermatitis chronica atrophicans, a unilateral ultraviolet discoloration of the extensor surfaces of the upper or lower limbs [12]. 

Lyme disease also manifests neurologically, most commonly in the United States as Bell’s palsy, a peripheral facial palsy presenting with facial paralysis on one or both facial hemispheres [13]. The spectrum of other neurological manifestations that appear in early disseminated Lyme disease includes radiculoneuritis, lymphocytic meningitis, and cranial neuropathy in the early disseminated stage [14]. 

Lyme arthritis can be a manifestation of both early and late disease dissemination. Early disseminated arthritis can manifest as arthralgias commonly found in one or both knees that come and go in a migratory pattern [15]. Late disseminated phase Lyme arthritis results in marked joint swelling, most commonly in the knees as well as the shoulder, ankle, elbow, and temporomandibular joint [16]. 

**Figure 2:**
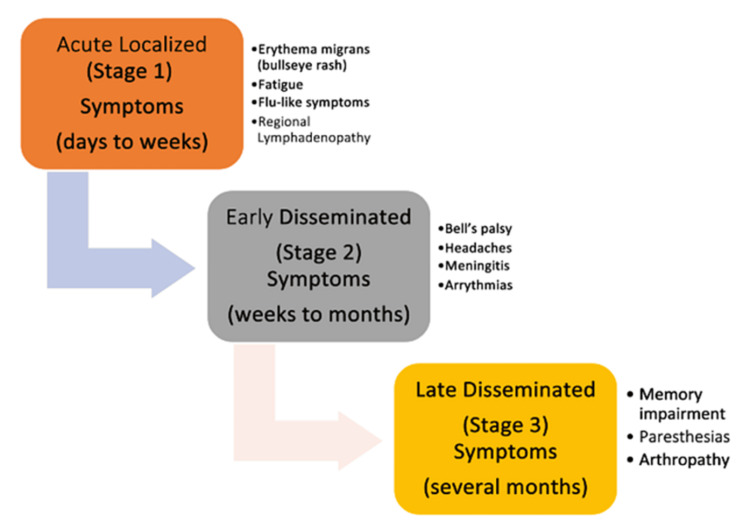
Systemic manifestations seen in Lyme disease Information gathered from [17,18]

Lyme carditis is an early disseminated Lyme disease manifestation that is characteristic of the disease. Lyme carditis has classic physical manifestations in the heart and pathologic electrical rhythms such as atrioventricular (AV) blocks. A summary of the findings is shown in Figure 2.

Lyme carditis manifestations

Lyme carditis is estimated to affect 1% of people with Lyme disease, a lower estimate than previous research, which is attributed to earlier recognition of classic Lyme disease presentations [19]. Lyme carditis is an early disseminated sign of Lyme disease affecting the heart and its accompanying tissues, manifesting most commonly as AV blocks of varying degrees [10]. The common signs and symptoms of Lyme carditis include light-headedness, fainting, shortness of breath, heart palpitations, and chest pain [20]. It is imperative for clinicians to be aware of the signs and symptoms of Lyme carditis, as it can be lethal and has been the cause of death in numerous patients between 1985 and 2019 [20]. Early treatment of Lyme carditis is crucial, as many of the symptoms and accompanying AV blocks resolve with the completion of the appropriate course of antibiotics [21]. Recent research has shown various atypical manifestations, suggesting a need for a comprehensive review of presentations to further educate physicians and increase their awareness of atypical displays.

## Review

Methods

All articles included in the review were collected from three databases: PubMed, MEDLINE, and CINAHL. The database search was conducted using the Boolean operators “AND” and “OR” between the keywords selected for the literature search as follows: (Lyme disease) AND (Carditis) OR (Heart Block) OR (Arrhythmia) OR (Heart failure). All years of publication were included in the initial screening. Article types included in the search were systematic reviews, literature reviews, and case reports, both full-length and published abstracts relating to cardiac manifestations of Lyme disease. All articles selected were published in the English language between the years 1980 and 2023. All articles included in the review focused on the cardiac manifestations of patients infected with Lyme disease at any stage of disease progression. Studies included participants ranging from early childhood to elderly adults. This literature review is limited by the amount of research currently available. A potential limitation of this literature review is the focus on literature that was published with the intent of highlighting only cardiac manifestations of Lyme carditis. Literature that highlighted other systemic manifestations and did not fully expand on possible atypical cardiac manifestations were screened out.

Results

Table 3 displays the findings of the literature review for the atypical manifestations of Lyme carditis.

**Table 3:** Atypical manifestations of Lyme carditis reported in case reports and cohort studies AV: atrioventricular, ECG: electrocardiogram, EMS: emergency medical services, IV: intravenous

Author	Type of study	Lyme disease cardiac manifestation	Significant findings
Steere et al. [22]	Retrospective cohort study	- Fluctuating degrees of AV block – Left ventricular dysfunction-Cardiomegaly	1) The study included 20 patients from the ages 6-58 years. 2) The study found 18 patients to have fluctuating degrees of AV heart block and all blocks appeared to be proximal to the bundle of His. 3) The study found 5 patients who developed left ventricular dysfunction. 4) The study found 1 patient who developed cardiomegaly. 5) The study determined AV blocks to be the common manifestation among patients.
Zainal et al. [23]	Case report	- Atrial fibrillation – Mobitz type 1 AV block	1) A 46-year-old male presented initially to the emergency department with atrial fibrillation. 2)Treatment with metoprolol was started and the patient reverted to sinus rhythm and was discharged. 3) Follow-up with primary care displayed a rash which was treated as cellulitis. 4) Presented to emergency department again with Mobitz type 1 AV block. 5) Lyme disease was diagnosed and treated with ceftriaxone.
Uzomah et al. [24]	Retrospective cohort analysis	- Myocarditis – Left bundle branch block – Right bundle branch block	1) The study analyzed the cardiac rhythms of over 100,000 hospitalized patients with Lyme disease. 2) Found an increased prevalence of myocarditis. 3) Found an increased prevalence of newly formed left and right bundle branch blocks in patients.
Paparone et al. [25]	Case report	- Complete heart block	1) A 32-year-old male presented with complete heart block as the initial presentation of Lyme carditis. 2) He was treated with antibiotics and rhythm resolution occurred.
Isha et al. [26]	Case report	- Complete heart block with AV dissociation – Bradycardia	1) A 21-year-old male presented with severe bradycardia. 2) Initial ECG demonstrated complete heart block with AV dissociation and diffuse T-wave abnormalities. 3) Developed into severe bradycardia and unresponsive to atropine. 4) Treatment with transcutaneous pacing and ceftriaxone was begun. 5) Resolution of heart block on follow-up with primary.
Myers et al. [27]	Case report	- Unprecedented symptoms of chest pain and palpitations	1) A 16-year-old male presented with acute onset of palpitations and chest pain. 2) The patient developed first-degree AV block. 3) The patient then developed a second-degree AV block. 4) Sinus rhythm was restored upon completion of IV antibiotic treatment.
Grewal et al. [28]	Case report	- Third-degree AV block	1) A 64-year-old female presented with a third-degree AV block. 2) Treatment with antibiotics did not lead to rhythm resolution. 3) A pacemaker was placed due to the failure of antibiotics to resolve abnormal rhythm.
CDC [29]	Case report series	- Sudden cardiac death	1) Two case reports of patients presenting to EMS after complaints of chest pain and collapse at home. 2) Both patients were pronounced dead at the hospital after sudden cardiac death. 3) Both patients had no past medical history and had no initial signs of Lyme disease on hospital presentation. 4) Both patients’ post-mortem evaluations showed pancarditis or myocarditis. 5) 1 case report of a male who had a prior 7-day history of shortness of breath and anxiety. 6) The patient was given anxiolytics after no rash, arthralgias, or neurologic symptoms were seen. 7) The patient presented 7 days later and was pronounced dead after sudden cardiac death. 8) Post-mortem evaluation showed signs of Lyme myocarditis.
Koene et al. [30]	Case report	- Acute heart failure	1) A 47-year-old female who presented with a complete heart block. 2) Treated with ceftriaxone with a resolution of heart block. 3) The patient then developed acute heart failure with an ejection fraction of 10%.
Malik et al. [31]	Case report	- Heart failure with mitral valve regurgitation	1) A 22-year-old male with Lyme disease presented with heart failure and a left ventricle ejection fraction of 49%. 2) Mild diffuse hypokinesis and moderate to severe mitral valve regurgitation were also seen. 3) Treatment with ceftriaxone was started. 4) Completion of treatment and follow-up evaluation showed resolution of heart failure and resolution of the mitral valve regurgitation.
Chaus et al. [32]	Case report	- Complete heart block with junctional escape rhythm – Paroxysmal atrial flutter – Short R-P tachycardia	1) A 61-year-old female presented with syncope and initial electrocardiogram rhythm of complete heart block with junctional escape rhythm and positive Lyme disease titers. 2) Cardiac MRI and transthoracic echocardiography revealed a structurally normal heart. 3) Treatment with ceftriaxone was begun. 4) On day 4 of admission the patient's rhythm developed into paroxysmal atrial flutter and short R-P tachycardia. 5) Antibiotics were completed and the patient was discharged with a first-degree AV block.
Tabot et al. [33]	Case report	- Left bundle branch block	1) A 62-year-old female presented with a 3-week duration of palpitations, exertional shortness of breath, atypical chest pain, skin rash, and positive Lyme disease titers. 2) ECG revealed a new left bundle branch block. 3) Treatment with ceftriaxone was started. 4) The patient showed resolution of the left bundle branch block on follow-up ECG after completion of ceftriaxone treatment.
Najam et al. [34]	Case report	- First-degree AV block – Mobitz type 1 second-degree AV block – Mobitz 2:1 block	1) A 70-year-old male presented with progressive orthopnea, dyspnea, and positive Lyme titers. 2) The patient’s initial ECG showed a type 1 AV block. 3) The patient then developed a Mobitz type 1 second-degree AV block with intermittent 2:1 block. 4) Treatment with ceftriaxone was started and the patient was discharged with doxycycline. 5) On follow-up with the primary care physician, the rhythm was found to be in normal sinus.

Lyme disease presents with various symptoms and possesses the ability to affect multiple organ systems. If the disease progresses due to lack of treatment, the effects tend to spread throughout the body to various organs. Of the various organs, the cardiac system is one of the more commonly affected organ systems by Lyme disease. Lyme carditis is a systemic manifestation that clinicians should be cognizant of due to the long-lasting deleterious effects and, at times, it can be the only symptom of Lyme disease. Increasing the understanding of Lyme carditis can allow for early detection and treatment without long-term effects.

*B. burgdorferi*, the main cause of Lyme disease, modulates the expression of its surface proteins, which can allow access to host tissues secondary to molecular mimicry. Proteins related to cardiac tropism include P66 and the decorin-binding proteins that allow Lyme disease to colonize and attack host tissues [35]. The spirochete infiltrates various locations of the heart, including the collagen fibers at the base of the heart, the interventricular septum, and the inner and outer membranes [19]. Once the myocardium has been colonized by *B. burgdorferi*, an immune response subsequently occurs, damaging the heart tissue and affecting both the muscle and electrocardiogram system of the heart [19]. 

**Figure 3:**
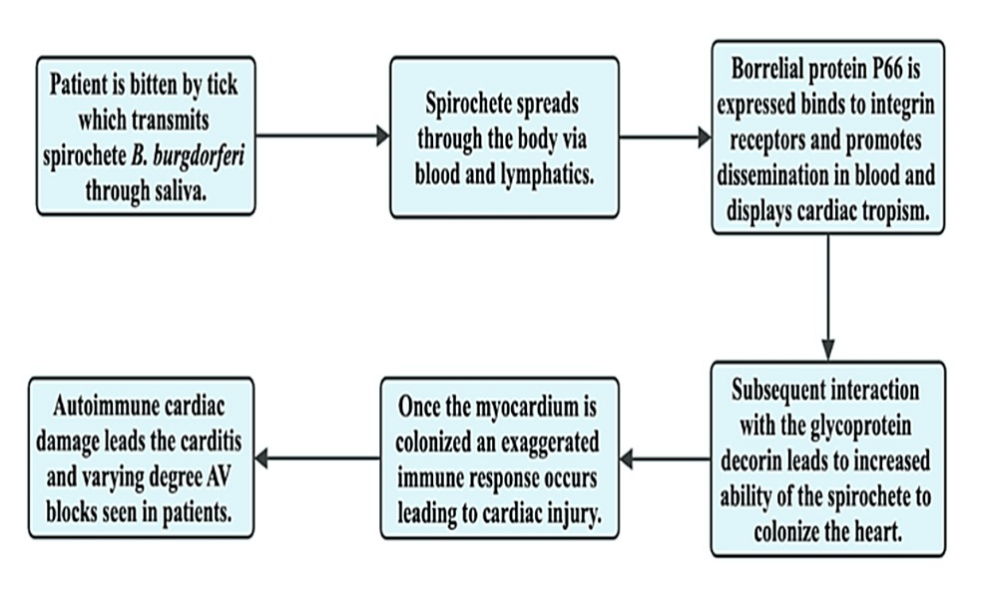
Pathogenesis of Lyme carditis Information gathered from [37]

Rotsoff et al. found in their primate research that the inflammation observed in Lyme carditis was primarily due to macrophages and lymphocytes, as opposed to the neutrophil-predominant inflammation seen with Lyme arthritis [36]. He also identified a relationship between the increased number of spirochetes found in the cardiac tissue and the degree of conduction disturbance and intensity of myocardial inflammation [36]. The myocardium can be affected independently, with the pericardium, or it can involve all layers of the heart leading to pancarditis seen in various patients [19]. Figure 3 depicts the proposed pathogenesis of inflammatory damage causing Lyme carditis.

The exact mechanism behind Lyme disease causing AV blocks has not been fully understood, but researchers have suggested that the autoimmune inflammation seen in patients leads to conduction abnormalities [38]. Kostic et al. proposed that the cross-reactive IgM antibodies seen in patients with Lyme disease can react with self-components leading to autoimmunity and are a possible cause of the conduction abnormalities observed [39]. He proposed that autoimmunity affects various areas and nodes of the heart, with the AV node being the most commonly affected, leading to the AV blocks seen in patients [39]. This proposed pathogenesis explains why Lyme disease has been known to induce electrical abnormalities resulting in AV blocks of varying degrees, ranging from first-degree to third-degree. Typically, these blocks can result in shortness of breath, dizziness, weakness, or chest pain [10]. 

Clinicians must recognize AV blocks early and document a detailed history from the patient as these blocks can be the only sign of Lyme disease. Paparone et al. describe a case of an asymptomatic patient with a high-degree AV block due to Lyme disease, ultimately resulting in the patient's death [25]. Other electrical abnormalities have been seen in patients with Lyme disease as well, either presenting on their own or stemming from an early AV block. Uzomah et al. describe multiple cases of Lyme disease affecting the electrical system of the heart, where patients only presented with asymptomatic new right or left bundle branch blocks [24]. Zainal et al. describe another case of a patient with Lyme disease who initially presented with atrial fibrillation [23]. Lyme disease manifestations of the heart can also lead to fatal outcomes, as seen by the CDC, which describes three cases of sudden cardiac death in patients who, upon arrival by emergency medical services, were found to be deceased with each of them showing evidence of Lyme disease. 

Given the various presentations of electrical abnormalities associated with Lyme disease, it is important for clinicians to remain vigilant in efforts to limit disease progression and avoid fatal outcomes associated with these pathologic rhythms. The vigilance of clinicians does not end with the electrocardial manifestations, as Lyme disease also affects the physical heart. Thus, clinicians should be aware of all manifestations in order to effectively diagnose and treat patients with Lyme carditis.

The early disseminated phase of Lyme disease can affect the heart and its accompanying tissues, causing Lyme carditis. Lyme disease is unique in that it can infect all parts of the heart, including the AV node, inner and outer heart membranes, the cardiac muscles themselves, blood vessels, and, rarely, the heart valves [40]. Physical manifestations, as opposed to the more common electrocardial manifestations of Lyme carditis, are less commonly seen but have been described in the literature. For instance, myocarditis was observed in a study by Uzomah et al., who looked at thousands of patients with Lyme disease and found many of them to have myocarditis [24]. Malik et al. describe a case of mitral regurgitation in a young 22-year-old patient. In this case, the valvular abnormality resolved after the patient was treated for and cured of Lyme disease [31]. This is a unique case as Lyme disease has not been commonly known to affect heart valves. Koene et al. describe a case of a patient who developed acute heart failure with an ejection fraction of 10% after treatment of Lyme disease [30]. In this case, the patient had an AV block, commonly found in Lyme disease, but heart failure with a reduced ejection fraction is rarely seen.

Therefore, due to the various manifestations of Lyme disease and its effects on both the physical heart and the electrical conduction system, it is important for clinicians to be aware of the varying symptoms that may present in order to appropriately diagnose and treat Lyme disease.

Lyme disease is commonly described by various characteristic symptomatic presentations that signify infection, such as erythema migrans rash, Bell’s palsy, and arthritis. Some of the additional common cardiac manifestations seen are AV blocks of varying degrees and possibly Lyme carditis. Although these symptomatic displays are characteristic of the disease, various unique cardiac manifestations have been described and do not align with the more commonly seen cardiac manifestations. With cases on the rise, it is important to educate clinicians on the unique and changing manifestations of Lyme disease, especially those affecting the heart. As noted in the review, patients can present asymptomatically with new arrhythmias or with new valvular abnormalities as the only manifestation of the disease. Therefore, clinicians must keep Lyme disease on their differential and be aware of all manifestations in order to identify and treat it effectively.

## Conclusions

Lyme disease is a progressive multi-system disease in which presenting symptomatology may vary depending on the degree and extent of the illness, especially if the condition progresses without treatment. Patients may present with erythema migrans as an early manifestation or cardiac symptoms as a late manifestation if left untreated. Due to the rise in the incidence of Lyme disease in the United States and the long-term complications associated with the disease, familiarization with various symptomatology and management options is warranted by clinicians. This review serves the purpose of increasing awareness among clinicians regarding the pathophysiology, mechanism of action, symptomatology, treatment, and management of Lyme disease to prevent unfavorable outcomes.
